# A Case of Profunda Femoris Artery Pseudoaneurysm Treated With Catheterization and Surgical Hematoma Removal

**DOI:** 10.7759/cureus.42708

**Published:** 2023-07-30

**Authors:** Nao Kume, Kensuke Konagaya, Takashi Okamoto, Hidemitsu Ogino

**Affiliations:** 1 Vascular Surgery, Narita Tomisato Tokushukai Hospital, Chiba, JPN; 2 Surgery, Narita Tomisato Tokushukai Hospital, Chiba, JPN; 3 Cardiovascular Surgery, Narita Tomisato Tokusyukai Hospital, Chiba, JPN

**Keywords:** proximal femoral fracture, hybrid surgery, hematoma removal, transcatheter arterial embolization, profunda femoris artery pseudoaneurysm

## Abstract

A pseudoaneurysm of the profunda femoris artery (PFA) is a rare disease induced by femoral trauma, proximal femoral fracture, or iatrogenic causes associated with orthopaedic surgery. Recently, transcatheter arterial embolism was reported as an effective treatment for profunda femoris artery pseudoaneurysm. This report presents the case of an 85-year-old male who underwent artificial head replacement for a left femoral neck fracture and was hospitalized with a peri-stem fracture four years later. Conservative treatment was conducted with a brace, though the swelling and pain in his left thigh increased one week after his hospital admission. Contrast-enhanced computed tomography (CT) led to a diagnosis of a left profunda femoris artery pseudoaneurysm, and the patient underwent emergency transcatheter arterial embolism and surgical hematoma removal. Since the emergency surgery, the patient’s course has been good, and he has been undergoing rehabilitation.

## Introduction

Profunda femoris artery (PFA) pseudoaneurysm is a rare disease that is caused by femoral trauma, proximal femoral fracture, or iatrogenic causes associated with orthopaedic surgery [1]. In recent years, transcatheter arterial embolization (TAE) has been considered an effective treatment for PFA pseudoaneurysms [2-4]. This report presents a case of PFA pseudoaneurysm caused by a femoral fracture that was treated with TAE.

## Case presentation

**Figure 1:**
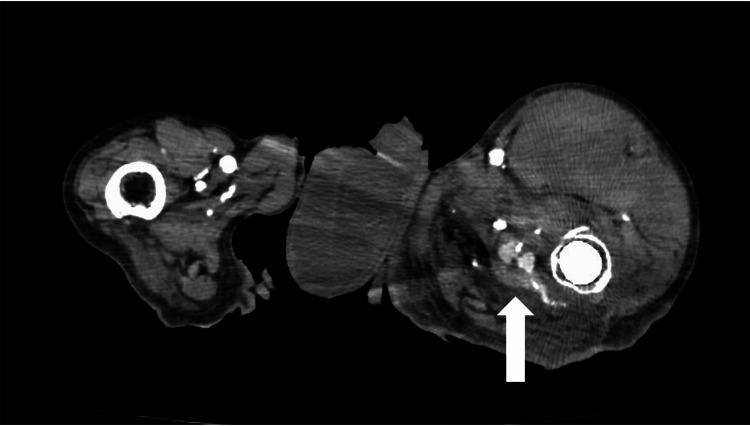
An axial CT scan This shows bleeding within the left thigh (arrow).

An 85-year-old man presented with a chief complaint of left thigh pain and a history of falling two weeks prior to the presentation. He had a medical history of diabetes, and he has left hemiplegia due to cerebral infarction. Four years ago, he underwent an artificial head replacement for a left femoral neck fracture. Computed tomography (CT) resulted in a diagnosis of peri-stem fracture after femoral head replacement, and cortical thinning was also observed. Surgical fixation was difficult. As the patient was already a left hemiplegic, conservative treatment with fixation using a brace was performed. Two weeks after the patient’s hospital admission, the pain and swelling in the left thigh worsened, and he was diagnosed with PFA haemorrhage based on the results of contrast-enhanced CT (Figure 1).

**Figure 2:**
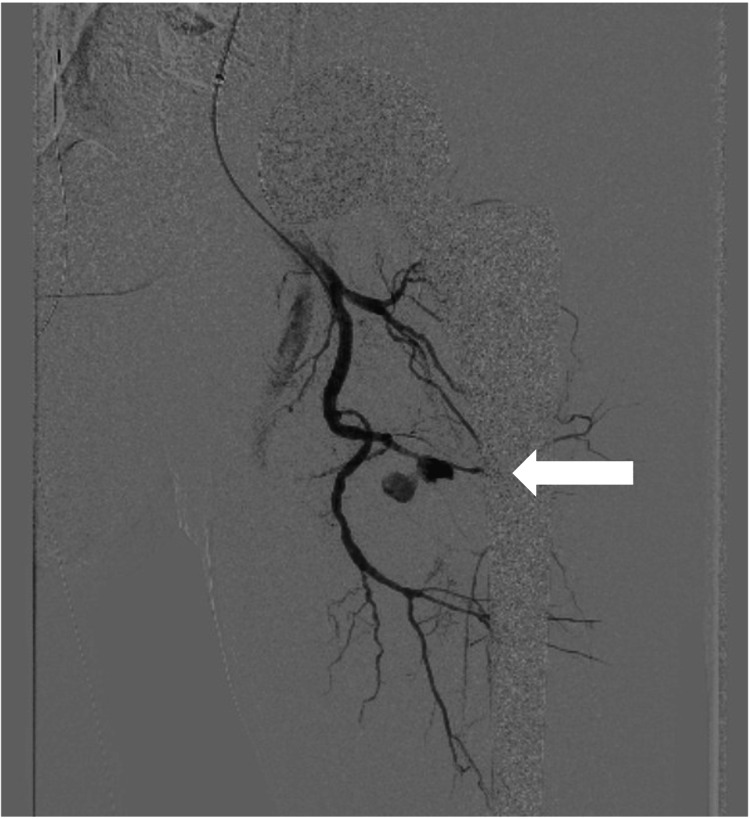
An angiography before coil embolization This shows a pseudoaneurysm was identified in the second branch of the PFA (arrow).

The patient also exhibited hypotension and was diagnosed with hemorrhagic shock. He underwent emergency angiography under general anaesthesia. A 5-Fr-long guiding sheath (DestinationR, Terumo, Tokyo, Japan) was inserted from the right common femoral artery to the left common femoral artery using a crossover approach. Angiography was conducted, and a pseudoaneurysm was identified in the second branch of the PFA (Figure 2).

**Figure 3:**
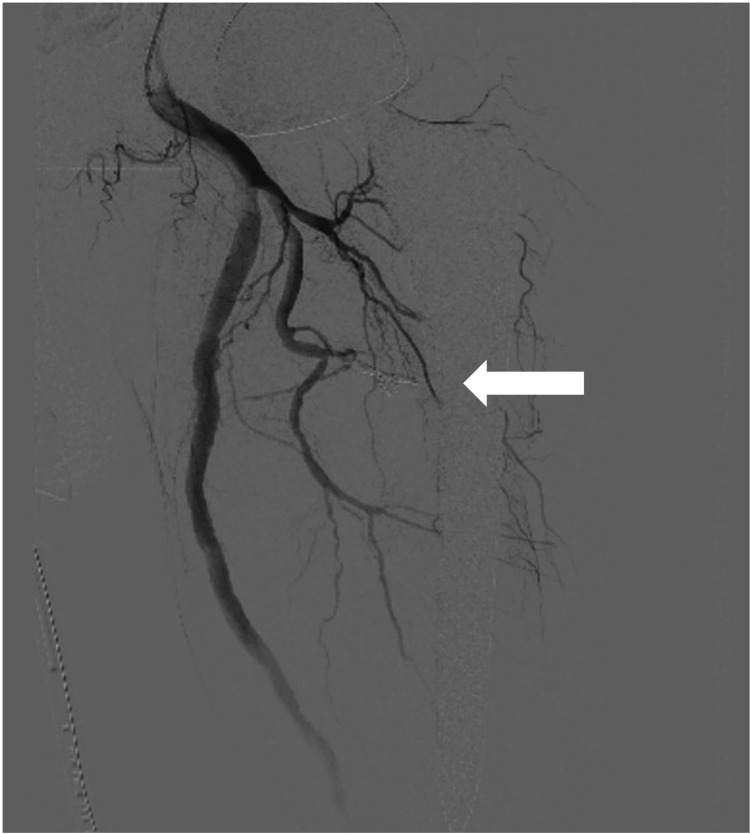
An angiography after coil embolization This shows that the pseudoaneurysm disappeared after embolization (arrow).

The feeding branch was selectively catheterized using a 2.0-Fr microcatheter (SniperR2, Terumo, Tokyo, Japan). It was confirmed that there were no outflow vessels from the pseudoaneurysm other than the main trunk, and coil embolization was conducted using the isolation method. Coils (Azur Soft 3DR [4 mm × 15 cm, 3 mm × 10 cm, 2 mm × 8 cm], Terumo, Tokyo) and Interlock Fibered IDCR (]4 mm × 8 cm, 2 mm × 4 cm], Boston Scientific, Massachusetts, USA) were used from the outflow vessels to the inflow vessels to conduct coil embolization. Angiography confirmed the disappearance of the pseudoaneurysm (Figure 3).

Before coil embolization, we inserted a needle into the muscle and connected the needle with the arterial line transducer in order to measure the internal pressure of the left anterior compartment of the left thigh. It was 55 mmHg. To avoid the risk of compartment syndrome and to prevent postoperative hematoma infection, an incision was made in the proximal thigh to remove the hematoma in the anterior compartment. The postoperative course was favourable, with no rebleeding or infection in the left thigh.

## Discussion

PFA pseudoaneurysms are rare and account for approximately 2% of peripheral arterial injuries [5]. Most PFA pseudoaneurysms are caused by iatrogenic factors during orthopaedic surgery, and a few cases are due to femoral fractures [6]. In the patient presented in this case, surgery was not conducted during this hospitalization, and the vascular injury was believed to have occurred due to femoral bone fragments. Sun et al. reported that the PFA is located within 13 mm of the femur and, therefore, has a high risk of injury [7]. As ageing societies continue to develop, femoral fractures are becoming more common, and the possibility of vascular injuries cannot be ignored. Pseudoaneurysms are diagnosed using ultrasound tests, computed tomography angiography (CTA), and angiography. CTA is capable of three-dimensionally reconstructing the vascular system of the lower limbs and has high sensitivity (90-95%) and specificity (98-100%) for detecting post-traumatic arterial injury. CTA is also a rapid and non-invasive diagnostic method [8].

Pseudoaneurysms can be treated using one of three methods. Ultrasound-guided thrombin injection was a successful treatment of femoral aneurysms in 1986 and was first used to treat PFA pseudoaneurysms in 1987 [9]. This method is safe and effective, with a success rate of 93%. However, large hematomas make it difficult to identify feeding vessels using ultrasound, and the cannulation of deep or small aneurysms is technically difficult. Therefore, this method is not suitable for all cases [9]. The second treatment method is surgery, including an incision to expose the artery through the hematoma to achieve hemostasis. This method is selected in cases of rupture, limb ischemia, compression neuropathy, and skin necrosis, and hematoma removal can also be performed. The incidence of postoperative complications is approximately 20%, and mortality is as high as 3% [10]. Catheter embolization can also be used to treat pseudoaneurysms, and this is the most effective treatment method for PFA pseudoaneurysms [2]. The endovascular approach is an important and effective surgical treatment option as it is minimally invasive, has a short recovery period, and is relatively painless.

In this report, TAE was performed on the second branch of the PFA to control bleeding from the pseudoaneurysm. The preoperative internal pressure of the anterior thigh compartment was 55 mmHg, indicating a high risk of thigh compartment syndrome. A subsequent groin incision was made to remove the anterior femoral compartment hematoma to prevent thigh compartment syndrome, hematoma infection, and delayed wound healing. Surgical hemostasis of PFA pseudoaneurysms has a high frequency of postoperative complications, and it is often difficult to identify the source of bleeding. Therefore, in the present case, catheter treatment and surgical hematoma removal were conducted, resulting in good wound healing and no local infection or rebleeding.

## Conclusions

PFA pseudoaneurysms are rare. In our case, a combined treatment of catheter embolization and surgical hematoma removal was conducted. This method is effective and reliable for controlling bleeding. Besides, the surgical removal of hematomas is also effective in promoting wound healing because the method can prevent compartment syndrome and local infection due to the hematoma.
